# Supplementary material for: Overexpression of Hepatocyte Chemerin-156 Lowers Tumor Burden in a Murine Model of Diethylnitrosamine-Induced Hepatocellular Carcinoma
Source: Int J Mol Sci. 2019 Dec 30;21(1):252. doi: 10.3390/ijms21010252 (PMC6982125; doi:10.3390/ijms21010252)
Supplement: Supplementary file 1 [file ijms-21-00252-s001.pdf]

Supplementary table 1: Genes which were upregulated more than 2-fold in tumors of Control-AAV infected mice were selected. Real-time RT-PCR analysis revealed that Spink1 is significantly induced in the tumors and the respective p-value for this difference (p = 0.01289) was chosen as cut off value. None of the genes differed in the normal liver tissues (NT) of Control-AAV and chemerin-156-AAV (Ch-156) (n = 5 mice) infected animals. Gm25482 (predicted gene, 25482) was reduced in tumor tissues (TT) of mice overexpressing chemerin-156 and is highlighted in red. Well established tumor markers (alpha fetoprotein, Afp; glypican 3, Gpc3) and genes analyzed by real-time RT-PCR or immunoblot analysis are highlighted in grey color. Fold changes (FC) and p-values for the comparison of TT and NT of Control-AAV and of chemerin-156-AAV injected mice, respectively, are given in the columns 1 to 4. Fold changes (FC) and p-values for comparison of NT or TT of both groups are listed in columns 5 to 8.

1	2	3	4	5	6	7	8	
FC TT/NT Control-AAV	p-value TT/NT Control-AAV	FC TT/NT Ch-156 AAV	p-value TT/NT Ch-156 AAV	FC NT Ch-156 /NT Control-AAV	p-value NT Ch-156 AAV /NT Control-AAV	FC TT Ch-156 AAV /TT Control-AAV	p-value TT Chemerin-156-AAV /TT Control-AAV	Gene Symbol
25.44	0.00399	21.04	0.00319	1.39	0.49125	1.15	0.82745	Akr1c18
17.28	0.00546	21.41	0.03110	-1.16	0.40119	1.07	0.95005	Gpc3
12.63	0.00362	8.56	0.03087	1.25	0.55302	-1.18	0.82850	Tspan8
11.38	0.00159	12.32	0.00743	1.05	0.84113	1.14	0.79193	Afp
9.48	0.00826	12.51	0.00684	-1.54	0.28641	-1.17	0.82601	Kif5c
9.33	0.00087	11.98	0.00032	-1.15	0.74079	1.11	0.81961	Mir675
8.14	0.00266	8.29	0.00283	-1.17	0.46269	-1.15	0.71303	Nrg1
7.87	0.00959	12.06	0.00818	-1.19	0.60191	1.29	0.69246	Slpi
7.28	0.00327	9.87	0.00807	-1.26	0.28350	1.07	0.91391	5330417C22Rik
7.09	0.00628	9.33	0.00080	1.10	0.79407	1.45	0.22688	---
6.62	0.00786	7.70	0.00072	1.14	0.71667	1.33	0.34079	Scd2
6.42	0.01111	17.26	0.00076	-1.29	0.41866	2.09	0.06010	Alox5
6.30	0.00404	4.72	0.02786	1.45	0.22628	1.09	0.89850	Bex1
5.66	0.00880	7.77	0.00003	-1.06	0.75199	1.30	0.49502	Cidea
5.65	0.00536	8.16	0.00683	-2.24	0.06389	-1.55	0.38009	Igfbp1
5.61	0.00386	4.73	0.00045	1.24	0.30465	1.04	0.86127	Sytl5
5.58	0.00240	5.98	0.00299	1.05	0.84861	1.13	0.78640	Sprr1a
5.57	0.00818	5.82	0.00063	-1.04	0.91561	1.01	0.96441	Ifi27l2b
5.53	0.01033	3.78	0.02286	-1.11	0.45506	-1.62	0.38111	Adamts14
5.37	0.00526	5.42	0.00321	-1.03	0.87135	-1.02	0.95340	Golm1
5.32	0.00369	7.05	0.00356	-1.17	0.57468	1.13	0.67683	Igdcc4
5.27	0.00220	7.24	0.00054	-1.36	0.17591	1.01	0.96070	Gria3
5.26	0.01289	10.48	0.01151	1.25	0.26944	2.49	0.15826	Spink1
5.24	0.00792	6.88	0.00497	1.02	0.94798	1.34	0.43597	Wfdc15b
5.22	0.01070	4.14	0.03108	1.03	0.82303	-1.23	0.71565	Krt20
5.11	0.00663	6.34	0.00206	-1.27	0.40083	-1.03	0.93732	LOC105245453
5.04	0.00569	7.19	0.00568	-1.08	0.77180	1.33	0.44206	Tlr1
4.97	0.00510	6.40	0.00217	-1.30	0.33974	-1.01	0.98432	Nid1
4.95	0.00332	5.34	0.00475	-1.33	0.13618	-1.23	0.62174	Krt23
4.88	0.00972	5.14	0.00205	-1.46	0.36554	-1.38	0.45471	Dhrs9
4.76	0.00039	6.10	0.00053	-1.39	0.14856	-1.08	0.62679	Cd63
4.74	0.00442	8.56	0.00007	-2.56	0.02399	-1.42	0.11209	Egr1
4.68	0.00146	4.75	0.01263	-1.12	0.65685	-1.11	0.78745	Il1rn
4.56	0.01266	7.20	0.00164	-1.47	0.05801	1.07	0.87031	Casc4

4.44	0.00730	5.59	0.00028	1.33	0.18687	1.68	0.15365	Pnpla3
4.44	0.00850	7.08	0.00031	-1.61	0.23391	-1.01	0.93984	Abcd2
4.39	0.00864	7.33	0.00017	-1.13	0.62900	1.47	0.07528	Pls1
4.24	0.00097	5.15	0.00030	-1.02	0.91702	1.20	0.27900	Ccnd1
4.10	0.00228	4.41	0.00052	1.11	0.69344	1.19	0.29249	Sqle
4.07	0.00843	4.29	0.00627	-1.39	0.21434	-1.33	0.34413	Dusp5
4.07	0.00679	5.49	0.00099	-1.24	0.48834	1.09	0.61943	Cdh1
4.03	0.00671	4.15	0.00599	1.02	0.96009	1.05	0.82464	Tubb2a
4.02	0.00177	4.10	0.01557	-1.08	0.70905	-1.06	0.83003	1810010H24Rik
3.85	0.00565	3.19	0.00688	1.59	0.25245	1.32	0.44637	Cyp2b13
3.84	0.00501	4.45	0.00460	-1.06	0.83090	1.09	0.70213	Cyp2b9
3.84	0.00206	5.18	0.00189	-1.80	0.04981	-1.34	0.06578	Esm1
3.81	0.00420	5.10	0.00078	-1.78	0.06109	-1.33	0.31321	Btg2
3.75	0.00159	4.75	0.00045	1.02	0.94697	1.29	0.44092	Tmc5
3.75	0.01052	5.01	0.00181	1.08	0.80314	1.45	0.05259	Klk1b4
3.71	0.00673	5.26	0.00042	-1.34	0.17652	1.06	0.81381	Mtmr11
3.71	0.00960	5.99	0.00098	-1.41	0.37341	1.14	0.35507	Prom2
3.68	0.00521	3.69	0.00118	-1.22	0.26598	-1.21	0.43165	Tmem71
3.67	0.01071	5.06	0.00554	1.07	0.66908	1.48	0.38331	Gprc5b
3.67	0.00495	3.75	0.00406	1.05	0.76441	1.07	0.85197	Aebp1
3.66	0.00526	2.65	0.11473	1.14	0.48548	-1.22	0.72843	Cxcl17
3.62	0.00380	4.16	0.01076	-1.12	0.57166	1.02	0.95249	Pde6c
3.58	0.00490	2.96	0.00114	1.34	0.26339	1.11	0.58150	Nsdhl
3.58	0.00054	5.02	0.00103	-1.21	0.25198	1.16	0.33649	Plxnb1
3.55	0.00168	3.78	0.01758	1.33	0.10386	1.42	0.36604	Prc1
3.52	0.00339	4.91	0.00002	-1.28	0.30713	1.09	0.32058	Pygb
3.50	0.01266	3.53	0.01236	1.01	0.96093	1.01	0.96718	Tmem45b
3.50	0.00633	4.33	0.00049	-1.27	0.34637	-1.03	0.82130	Gm10680
3.48	0.00791	3.74	0.00063	-1.14	0.48715	-1.07	0.64560	Slc9a7
3.47	0.00817	3.15	0.00023	1.05	0.85822	-1.05	0.82508	Hmgcs1
3.42	0.00426	5.20	0.00005	-1.56	0.06825	-1.03	0.91355	Nipal1
3.38	0.01121	5.26	0.00046	-1.28	0.38932	1.21	0.09952	BC089597
3.36	0.00713	3.40	0.00067	1.25	0.29582	1.27	0.29278	Lss
3.35	0.01012	4.64	0.00033	-1.21	0.44326	1.15	0.50467	---
3.35	0.00619	3.68	0.00797	-1.06	0.67225	1.03	0.92376	Osbpl10
3.30	0.01214	4.05	0.00661	-1.24	0.26237	-1.01	0.97231	Tnfrsf10b
3.29	0.00651	2.57	0.00803	-1.08	0.73928	-1.39	0.13068	Ccdc120
3.29	0.00384	4.65	0.00120	-1.03	0.90831	1.38	0.20225	Cyp2a4
3.24	0.01249	5.50	0.00094	-1.19	0.40401	1.43	0.26011	Mki67
3.16	0.00077	2.82	0.00265	-1.22	0.39334	-1.37	0.14250	Cd1d2
3.14	0.00352	5.10	0.00008	-1.64	0.06161	-1.01	0.92824	Shc2
3.14	0.00518	3.54	0.00159	-1.22	0.31485	-1.09	0.66926	---
3.13	0.00524	4.38	0.00132	-1.10	0.49283	1.27	0.21961	Tstd1
3.13	0.00535	2.83	0.00111	1.21	0.42354	1.10	0.55189	Dhcr7
3.12	0.00101	3.09	0.06495	-1.68	0.33159	-1.70	0.32646	Cyp17a1
3.10	0.00581	2.04	0.05196	1.12	0.50266	-1.36	0.38330	A730090H04Rik
3.08	0.00249	3.18	0.00341	1.16	0.22373	1.19	0.59604	Ect2
3.07	0.00226	3.23	0.00113	-1.11	0.64397	-1.05	0.67991	Syne1
3.07	0.00595	3.40	0.00186	-1.30	0.27521	-1.17	0.43306	Dab1
3.05	0.00247	2.49	0.00311	-1.12	0.60201	-1.37	0.12904	Grhl1
3.04	0.00192	2.97	0.00063	-1.01	0.94785	-1.04	0.82917	Tnfsfm13
3.02	0.00371	3.30	0.00365	-1.23	0.14928	-1.12	0.60117	Bco1
2.98	0.01281	2.93	0.00228	-1.18	0.12996	-1.20	0.51767	Nlrp12
2.98	0.00453	3.42	0.00370	-1.05	0.71403	1.09	0.62929	Ces2e
2.95	0.00643	3.16	0.00736	-1.27	0.38125	-1.18	0.65539	Cntnap1

2.93	0.00289	3.36	0.00335	1.23	0.27331	1.41	0.19861	Lrrc24
2.93	0.00700	3.68	0.00731	-1.06	0.72925	1.19	0.65042	Col6a6
2.90	0.00007	2.74	0.00087	1.04	0.81094	-1.02	0.86869	Syne1
2.90	0.00014	3.32	0.00511	-1.04	0.81259	1.11	0.62314	Plek2
2.90	0.00887	1.62	0.05397	1.09	0.71533	-1.63	0.16079	Gm25923
2.89	0.00415	3.24	0.00229	-1.03	0.88920	1.09	0.66784	Fkbp11
2.86	0.00408	3.05	0.00106	-1.01	0.95816	1.05	0.59039	Syne1
2.86	0.00457	3.09	0.00118	-1.02	0.89925	1.05	0.76861	Fndc3b
2.86	0.00020	3.07	0.00004	-1.08	0.55760	-1.00	0.97680	Syne1
2.85	0.00435	3.99	0.00001	-1.18	0.38432	1.18	0.06465	Rbp1
2.85	0.00590	3.67	0.00040	-1.21	0.32915	1.06	0.56309	Sectm1a
2.85	0.00714	3.71	0.00086	-1.07	0.53135	1.22	0.49360	Ttc39aos1
2.85	0.00852	3.06	0.00168	-1.03	0.88882	1.05	0.83110	Tgfbr2
2.85	0.00442	2.57	0.01265	-1.23	0.05575	-1.37	0.29487	Tc2n
2.84	0.00381	2.46	0.07184	1.12	0.83050	-1.03	0.96090	Lcn2
2.84	0.00065	2.28	0.00037	1.18	0.25392	-1.06	0.62650	Syne1
2.83	0.00667	3.49	0.00889	-1.14	0.38092	1.08	0.81571	Sparc
2.83	0.00762	2.73	0.00032	-1.10	0.62061	-1.13	0.47771	Abi2
2.82	0.00362	3.97	0.00020	-1.29	0.16073	1.09	0.59223	P2rx7
2.81	0.01167	3.53	0.00187	1.29	0.23676	1.62	0.06668	Hao2
2.79	0.01244	2.58	0.00844	-1.09	0.63877	-1.18	0.51874	Pck2
2.79	0.00521	2.99	0.00025	1.24	0.14252	1.33	0.23298	Hmgcr
2.79	0.00837	2.65	0.00229	1.19	0.43544	1.13	0.57971	1810055G02Rik
2.78	0.00500	3.72	0.00729	-1.05	0.85811	1.27	0.35776	Chrm3
2.75	0.00427	3.31	0.00297	1.04	0.72907	1.25	0.30953	Drc1
2.74	0.00130	3.28	0.00116	-1.14	0.56910	1.05	0.82648	Cyp51
2.73	0.00461	1.80	0.32787	1.60	0.02687	1.06	0.92260	Cxcl13
2.72	0.00110	2.97	0.01092	-1.12	0.35119	-1.02	0.91463	Uggt2
2.71	0.00681	4.14	0.00251	1.11	0.47649	1.69	0.18556	Prom1
2.71	0.01108	2.38	0.04939	1.15	0.29762	1.00	0.98894	Ptpre
2.70	0.00236	3.80	0.00239	-1.39	0.01711	1.01	0.96689	Gm19510
2.70	0.00009	2.70	0.00023	-1.09	0.49703	-1.09	0.29103	Syne1
2.70	0.00215	3.14	0.00018	-1.23	0.18138	-1.05	0.56573	Syne1
2.69	0.00429	2.62	0.00501	-1.14	0.54415	-1.17	0.61698	Fgl2
2.69	0.00138	3.07	0.00090	-1.06	0.68778	1.08	0.60295	Syne1
2.67	0.00404	3.91	0.00713	-1.29	0.43775	1.13	0.25464	Serpina12
2.64	0.01201	2.56	0.04052	1.00	0.99949	-1.03	0.90248	Cd276
2.64	0.00030	3.52	0.00008	-1.25	0.18572	1.07	0.46505	Syne1
2.63	0.00065	3.28	0.00030	-1.23	0.21051	1.01	0.83077	Syne1
2.61	0.01184	7.38	0.00318	1.29	0.19409	3.65	0.01343	Pnpla5
2.60	0.00166	3.18	0.00011	-1.21	0.30283	1.01	0.84652	Syne1
2.60	0.00203	2.48	0.00463	1.33	0.27973	1.27	0.26945	Fdps
2.58	0.01075	3.02	0.00078	-1.12	0.49795	1.04	0.83180	Scn1b
2.58	0.00021	2.32	0.05149	-1.08	0.64020	-1.20	0.62962	Bcl2
2.56	0.00450	2.11	0.02740	1.03	0.82462	-1.18	0.54777	Panx1
2.56	0.00057	2.20	0.00071	1.13	0.25715	-1.03	0.87465	D1Ertd622e
2.56	0.00110	2.37	0.00046	1.05	0.81045	-1.02	0.91541	Idi1
2.55	0.00426	2.65	0.00012	1.18	0.44654	1.22	0.35340	Rdh11
2.55	0.01002	2.53	0.00855	-1.07	0.78971	-1.08	0.79481	Ifi44
2.54	0.00364	5.26	0.00012	-1.36	0.12519	1.52	0.03004	Col4a3
2.53	0.00542	2.37	0.02291	1.54	0.18685	1.45	0.14580	Fasn
2.53	0.00088	3.23	0.00123	-1.32	0.45630	-1.03	0.94376	Cxcl14
2.53	0.00598	2.76	0.01375	-1.26	0.15677	-1.15	0.51086	Id1
2.53	0.00129	3.22	0.00635	-1.12	0.31415	1.14	0.67060	Ckap2
2.52	0.00143	2.37	0.00343	1.09	0.78019	1.02	0.92576	Mpeg1
2.49	0.01225	3.53	0.00472	-1.08	0.42244	1.31	0.49198	Psat1

2.49	0.00015	2.41	0.00279	-1.01	0.95625	-1.04	0.77526	Syne1
2.48	0.01030	4.14	0.00073	-1.45	0.23410	1.15	0.25587	Tff3
2.48	0.00527	2.61	0.00740	1.16	0.31821	1.22	0.39705	Kif20a
2.47	0.00039	2.30	0.00458	-1.08	0.54886	-1.16	0.28214	Syne1
2.46	0.00301	2.57	0.00163	-1.00	0.97237	1.04	0.83044	Ttc39a
2.46	0.00404	2.72	0.00014	-1.29	0.08464	-1.17	0.17927	Sstr2
2.44	0.00239	2.22	0.00267	-1.04	0.81464	-1.14	0.46629	Gm13657
2.44	0.00375	2.43	0.00342	1.02	0.86775	1.02	0.92979	Syne1
2.43	0.00898	2.22	0.02513	-1.00	0.95846	-1.10	0.71078	C330027C09Rik
2.39	0.01170	2.08	0.00791	1.03	0.84252	-1.12	0.59136	Dusp6
2.37	0.00856	2.85	0.00090	-1.04	0.60250	1.16	0.44616	Rims2
2.35	0.00418	1.92	0.02767	1.03	0.93612	-1.19	0.44025	Angptl8
2.34	0.00544	2.41	0.01138	-1.02	0.89537	1.01	0.96252	Pygo1
2.34	0.00618	3.34	0.00000	-1.53	0.12336	-1.07	0.63860	Lpl
2.34	0.00002	2.73	0.00386	-1.04	0.78448	1.12	0.53171	Acsl4
2.33	0.01221	2.59	0.00116	-1.04	0.81512	1.07	0.71937	Myadm
2.33	0.00663	2.30	0.01347	1.52	0.09353	1.50	0.10185	Slc51b
2.32	0.00238	2.51	0.00241	-1.19	0.32425	-1.10	0.24304	Syne1
2.32	0.00788	2.21	0.00507	-1.25	0.12526	-1.31	0.08438	Col4a5
2.32	0.00531	2.48	0.00113	-1.05	0.84889	1.02	0.81782	Syne1
2.32	0.00412	2.42	0.01381	-1.00	0.97852	1.04	0.88531	Msantd3
2.31	0.00010	2.56	0.00046	1.16	0.27000	1.28	0.05813	Syne1
2.31	0.00140	3.32	0.00127	-1.33	0.04927	1.09	0.63340	D17H6S56E-5
2.29	0.00224	2.46	0.01173	-1.05	0.78510	1.03	0.85029	Syne1
2.29	0.00792	2.13	0.00242	1.26	0.25706	1.17	0.61025	Glipr1
2.29	0.00247	2.24	0.00074	1.05	0.64400	1.03	0.77947	Syne1
2.28	0.00106	2.41	0.00215	1.04	0.74080	1.10	0.40822	Syne1
2.28	0.00001	2.56	0.00030	-1.10	0.43671	1.02	0.91231	4930527F14Rik
2.26	0.00030	2.82	0.00032	-1.26	0.07586	-1.01	0.85464	Syne1
2.26	0.00434	2.67	0.00172	1.08	0.58211	1.27	0.27932	D430020J02Rik
2.25	0.00217	1.74	0.03381	1.24	0.44307	-1.05	0.89971	BC023105
2.25	0.00443	2.36	0.00051	-1.06	0.67377	-1.01	0.90667	Syne1
2.25	0.00041	1.98	0.03087	1.28	0.23161	1.13	0.65862	Abcb1a
2.25	0.00007	3.22	0.00007	-1.31	0.03526	1.10	0.17600	Syne1
2.24	0.00809	2.74	0.00300	-1.59	0.13744	-1.30	0.17454	Fabp4
2.22	0.00513	2.15	0.00223	-1.00	0.98436	-1.04	0.83297	Cers6
2.21	0.00308	2.23	0.00315	-1.13	0.23301	-1.12	0.46074	App
2.20	0.01060	2.22	0.00519	-1.40	0.04710	-1.39	0.12260	Cldnd2
2.19	0.01230	3.32	0.00096	-1.39	0.07034	1.08	0.77314	Gm11963
2.19	0.00173	2.46	0.00138	-1.01	0.91821	1.11	0.21879	Acsl5
2.17	0.00496	2.88	0.00094	-1.32	0.13027	1.00	0.98771	Syne1
2.17	0.00073	2.65	0.01392	-1.05	0.76818	1.16	0.64459	Cd40
2.17	0.00221	2.79	0.00188	-1.10	0.50262	1.17	0.21904	Syne1
2.17	0.00172	1.93	0.00055	1.21	0.39073	1.08	0.68241	Msmo1
2.17	0.00242	2.53	0.00424	-1.19	0.35549	-1.02	0.87703	Syne1
2.16	0.00726	2.41	0.00045	-1.02	0.83487	1.09	0.28546	Syne1
2.15	0.00252	1.98	0.00281	1.13	0.21319	1.03	0.79711	Acaca
2.15	0.00000	1.88	0.00563	-1.02	0.91873	-1.16	0.45165	Bmp8b
2.14	0.00945	2.05	0.03070	1.14	0.45758	1.10	0.76471	Arhgef2
2.13	0.00226	2.01	0.01952	-1.03	0.78419	-1.09	0.64392	Jazf1
2.12	0.00631	1.71	0.00675	-1.09	0.56914	-1.35	0.19266	Morc4
2.12	0.00330	2.17	0.00109	-1.03	0.81599	-1.01	0.95537	Plscr1
2.11	0.01042	1.66	0.01701	1.34	0.10152	1.05	0.73185	Mvk
2.11	0.00122	2.70	0.00138	1.08	0.40893	1.39	0.03611	Ccne1
2.10	0.00280	1.76	0.02750	-1.03	0.85626	-1.22	0.16046	Trim68
2.09	0.00229	2.29	0.00069	-1.11	0.45093	-1.01	0.91955	Ccdc125

2.09	0.00708	2.04	0.00333	1.01	0.96393	-1.02	0.91074	Pgm3
2.09	0.00182	2.61	0.00001	-1.18	0.24614	1.06	0.57253	Syne1
2.07	0.00212	2.71	0.00029	-1.04	0.84489	1.26	0.33705	Sel1l3
2.06	0.00498	1.86	0.00171	-1.04	0.86365	-1.15	0.17842	Nab2
2.06	0.00915	-1.04	0.86786	-1.17	0.46355	-2.51	0.00883	Gm25482
2.06	0.00153	2.68	0.00782	-1.33	0.28086	-1.02	0.88233	Osbpl3
2.06	0.01138	2.10	0.08383	1.13	0.10269	1.16	0.72219	---
2.06	0.01138	2.10	0.08383	1.13	0.10269	1.16	0.72219	---
2.06	0.00045	2.16	0.01125	-1.14	0.23379	-1.09	0.55350	Syne1
2.05	0.00027	2.77	0.00079	-1.31	0.07519	1.03	0.78764	Adrbk2
2.05	0.00076	2.39	0.00192	-1.10	0.49969	1.06	0.54019	Syne1
2.04	0.00706	2.58	0.00226	-1.05	0.67034	1.20	0.52570	Unc5b
2.03	0.00170	2.12	0.00209	-1.32	0.03744	-1.26	0.10143	Fam3c
2.03	0.00869	2.29	0.01651	-1.19	0.07912	-1.05	0.83391	Kif20b
2.03	0.00844	2.47	0.00573	-1.23	0.43715	-1.01	0.97156	Fam198a
2.03	0.00744	2.50	0.01109	-1.15	0.14610	1.08	0.78860	Rhbdf1
2.03	0.01069	2.06	0.00372	1.06	0.66981	1.07	0.62570	Satb2
2.02	0.00038	2.33	0.00085	-1.15	0.20475	1.00	0.96714	Syne1
2.01	0.00707	2.13	0.00143	-1.04	0.75232	1.02	0.90702	Ctps2
2.01	0.00804	2.66	0.00014	1.05	0.79932	1.39	0.07259	Prss8
2.01	0.00576	1.94	0.01857	1.12	0.22800	1.08	0.75265	Cenpf
2.01	0.00380	2.04	0.00964	-1.23	0.21305	-1.21	0.19699	Sorbs2

Supplementary table 2: Genes which were downregulated more than 2-fold in tumors of Control-AAV infected mice were selected. Real-time RT-PCR analysis revealed that Spink1 is significantly induced in the tumors and the respective p-value for this difference (p = 0.01289) was chosen as cut off value. Three genes (dedicator of cytokinesis 8, Dock8; phospholysine phosphohistidine inorganic pyrophosphate phosphatase, Lhpp and cytochrome P450, family 1, subfamily a, polypeptide 2, Cyp1a2) were higher in the normal liver tissues (NT) of Chemerin-156-AAV infected and are highlighted in red color. None of the genes differed in the tumor tissues (TT) of Control-AAV and chemerin-156-AAV (Ch-156) infected animals. Fold changes (FC) and p-values for the comparison of TT and NT of Control-AAV and of chemerin-156-AAV injected mice are given in the columns 1 to 4. Fold changes (FC) and p-values for comparison of NT or TT of both groups are listed in columns 5 to 8.

1	2	3	4	5	6	7	8	
FC TT/NT Control-AAV	p-value TT/NT Control-AAV	FC TT/NT Ch-156 AAV	p-value TT/NT Ch-156 AAV	FC NT Ch-156 /NT Control-AAV	p-value NT Ch-156 AAV /NT Control-AAV	FC TT Ch-156 AAV /TT Control-AAV	p-value TT Chemerin-156-AAV /TT Control-AAV	Gene Symbol
-2.01	0.01095	-2.55	0.00205	1.00	0.97522	-1.27	0.24709	Sugct
-2.01	0.01287	-2.21	0.00424	1.03	0.89327	-1.07	0.73382	Dkk4
-2.02	0.00125	-2.21	0.00721	1.02	0.84789	-1.07	0.68931	Notum
-2.02	0.01117	-2.61	0.04197	1.23	0.61767	-1.05	0.81972	Rnase2a
-2.02	0.00362	-2.06	0.03248	-1.89	0.13729	-1.93	0.10902	Gadd45g
-2.02	0.01081	-1.94	0.00998	-1.11	0.52547	-1.07	0.78060	Gpcpd1
-2.03	0.01281	-2.55	0.00090	1.15	0.41108	-1.10	0.43528	Lgr5
-2.03	0.01242	-2.32	0.00365	1.21	0.12257	1.06	0.78629	Rgn
-2.03	0.00691	-2.37	0.00191	1.30	0.08740	1.12	0.56421	Alas2
-2.05	0.00518	-2.68	0.00435	1.19	0.05141	-1.10	0.71693	Slco2b1
-2.05	0.00088	-1.31	0.06263	-1.37	0.04289	1.14	0.45160	Gm25149
-2.05	0.00248	-3.94	0.00112	1.14	0.48967	-1.69	0.02487	Acss3
-2.06	0.01014	-1.63	0.06444	-1.30	0.10434	-1.03	0.90283	Hnf1aos1
-2.06	0.00242	-2.44	0.00165	1.09	0.42356	-1.08	0.62431	Aox1
-2.07	0.01134	-2.21	0.00316	1.00	0.98236	-1.07	0.73721	Slco1b2
-2.09	0.00331	-2.13	0.00141	1.07	0.67014	1.05	0.80423	Lama3
-2.09	0.00011	-2.11	0.00093	1.01	0.87101	1.01	0.97277	Ptk2b
-2.12	0.00086	-2.26	0.01434	1.04	0.73763	-1.03	0.91672	Keg1
-2.12	0.00060	-2.52	0.00260	1.17	0.23074	-1.01	0.94405	Cyp2d9
-2.14	0.00050	-2.15	0.03795	1.18	0.41306	1.17	0.39369	Gabrb3
-2.14	0.00041	-2.62	0.00625	1.25	0.09869	1.02	0.87878	Hhip
-2.14	0.01006	-2.74	0.00304	1.17	0.09143	-1.09	0.71640	Slc25a21
-2.15	0.01104	-2.02	0.00638	-1.29	0.31908	-1.21	0.42128	Chrna2
-2.18	0.00509	-2.25	0.00126	1.19	0.00808	1.15	0.46488	Dock8
-2.24	0.00541	-2.63	0.00709	-1.13	0.62739	-1.32	0.34429	Ddc
-2.24	0.00100	-4.09	0.00006	1.63	0.01158	-1.12	0.28445	Lhpp
-2.24	0.00218	-2.78	0.00037	-1.01	0.91561	-1.25	0.30910	Avpr1a
-2.25	0.00998	-2.73	0.00427	-1.19	0.27430	-1.45	0.30897	Cyp8b1
-2.26	0.00499	-1.96	0.00286	-1.09	0.66019	1.05	0.84248	Ankrd55
-2.28	0.00140	-2.71	0.00112	1.02	0.90990	-1.17	0.52278	Colec10
-2.28	0.00473	-2.87	0.00189	-1.09	0.74200	-1.38	0.31936	Cyp7a1
-2.28	0.00431	-2.72	0.00034	1.10	0.10281	-1.09	0.60957	Cyp27a1
-2.29	0.00035	-2.39	0.01951	1.11	0.50774	1.06	0.63917	Cd163
-2.29	0.00036	-2.42	0.00311	1.02	0.94644	-1.04	0.84494	Mustn1

-2.29	0.00204	-2.57	0.01544	1.29	0.04135	1.15	0.54297	D630033O11Rik
-2.30	0.00255	-2.91	0.02471	-1.01	0.95593	-1.28	0.58444	Ces1e
-2.30	0.00949	-2.48	0.00613	1.13	0.03096	1.04	0.86282	Ces1b
-2.31	0.00249	-1.82	0.06268	-1.15	0.43040	1.11	0.69743	Serpinb1a
-2.32	0.00613	-3.54	0.00002	1.34	0.04654	-1.14	0.59290	Ildr2
-2.32	0.00402	-2.53	0.00467	1.09	0.34148	1.00	0.99961	Sulf2
-2.32	0.00061	-2.50	0.00768	1.09	0.65673	1.01	0.90227	Pcp4l1
-2.35	0.00158	-3.12	0.00011	1.24	0.14233	-1.08	0.57940	Tbx3
-2.35	0.01164	-1.63	0.01264	-1.02	0.93374	1.41	0.22715	Gm17077
-2.36	0.00915	-2.31	0.04516	1.06	0.88480	1.08	0.50280	E030018B13Rik
-2.38	0.00503	-2.56	0.00698	1.10	0.55709	1.02	0.92935	Sult5a1
-2.42	0.00815	-2.40	0.00931	-1.12	0.40467	-1.11	0.64168	Asic5
-2.42	0.00808	-1.94	0.17190	-1.48	0.39195	-1.18	0.55211	5730420D15Rik
-2.42	0.00042	-3.45	0.00014	1.11	0.53315	-1.28	0.07720	Plekhb1
-2.43	0.00364	-2.64	0.00207	1.09	0.74253	1.00	0.99492	Nr1i3
-2.44	0.00924	-2.78	0.00316	1.44	0.15128	1.26	0.23538	Fam25c
-2.44	0.00063	-2.79	0.00073	1.11	0.36924	-1.03	0.83652	Npr2
-2.45	0.00433	-3.28	0.00076	-1.13	0.63468	-1.51	0.08022	Csad
-2.47	0.00026	-2.72	0.00047	-1.09	0.63207	-1.20	0.26495	Serpine2
-2.48	0.00210	-2.80	0.01016	1.13	0.45854	-1.00	0.99757	Onecut2
-2.48	0.00164	-2.88	0.01958	-1.06	0.61989	-1.23	0.58014	Slco1a1
-2.48	0.00032	-2.98	0.00007	1.16	0.47161	-1.04	0.84429	Car1
-2.49	0.00726	-2.76	0.00250	1.05	0.80933	-1.06	0.86591	Cyp46a1
-2.54	0.00856	-2.49	0.00748	1.13	0.25422	1.15	0.67317	Cyp2c29
-2.56	0.00258	-2.96	0.00128	1.19	0.00965	1.03	0.89223	Cyp1a2
-2.57	0.00015	-2.94	0.00021	1.40	0.17143	1.22	0.48628	Hsd3b4
-2.57	0.00015	-2.94	0.00021	1.40	0.17143	1.22	0.48628	Hsd3b4
-2.58	0.00024	-2.06	0.00387	1.02	0.86681	1.27	0.20390	Lrit2
-2.60	0.00167	-2.55	0.00257	1.20	0.37070	1.22	0.26159	Rmdn2
-2.64	0.00566	-2.71	0.00119	1.00	0.99139	-1.02	0.91760	Kcnk1
-2.66	0.00130	-3.23	0.00095	1.08	0.68100	-1.13	0.56258	Ablim3
-2.69	0.00229	-3.42	0.00150	1.22	0.08659	-1.04	0.80923	Gas1
-2.73	0.00237	-1.79	0.00039	-2.31	0.04210	-1.52	0.14024	Hspa1b
-2.74	0.00220	-3.20	0.00018	1.40	0.01848	1.20	0.09203	Arhgef37
-2.75	0.00155	-3.12	0.00083	1.06	0.73550	-1.07	0.79962	9130409I23Rik
-2.75	0.00315	-3.82	0.00434	1.19	0.42574	-1.17	0.37256	Slc39a2
-2.79	0.00002	-3.33	0.00223	1.03	0.88895	-1.16	0.51945	Gna14
-2.81	0.00806	-2.60	0.00671	-1.21	0.47802	-1.12	0.59654	Ranbp3l
-2.84	0.01026	-2.98	0.01396	1.68	0.20203	1.60	0.21888	Gm12718
-2.87	0.00996	-3.13	0.02178	-1.09	0.81350	-1.18	0.77600	Gm13773
-2.90	0.00334	-3.49	0.01719	1.18	0.33884	-1.02	0.95759	Cyp2c50
-2.90	0.00509	-2.99	0.00814	1.02	0.87581	-1.01	0.97636	Scnn1a
-2.94	0.00036	-3.65	0.00731	-1.63	0.35716	-2.03	0.09933	Acot1
-2.95	0.00003	-3.21	0.00003	1.20	0.13923	1.11	0.34780	Slc15a5
-2.96	0.00102	-2.99	0.00012	-1.00	0.97287	-1.02	0.92693	Gm3776
-2.96	0.00578	-3.90	0.00265	1.18	0.28242	-1.11	0.69288	Pdilt
-2.99	0.00950	-3.61	0.00744	1.29	0.74799	1.07	0.92471	Saa2
-3.01	0.00123	-3.22	0.00017	1.19	0.03400	1.11	0.45670	Prodh
-3.02	0.00716	-2.28	0.03246	-1.02	0.95404	1.29	0.25014	Gm38455
-3.05	0.00092	-3.28	0.00023	-1.07	0.72751	-1.15	0.23400	Sema3e
-3.11	0.00616	-3.51	0.00440	-1.02	0.85056	-1.15	0.68598	Cyp4a12a
-3.14	0.00114	-3.51	0.00175	1.11	0.42949	-1.00	0.97935	Slc1a2
-3.27	0.00669	-4.11	0.00336	1.12	0.65787	-1.12	0.33181	Ctcflos
-3.28	0.00411	-4.18	0.00724	1.33	0.04510	1.04	0.88422	Oat
-3.34	0.00830	-3.66	0.00394	1.09	0.57856	-1.00	0.99101	Cyp2c54
-3.40	0.00102	-3.86	0.00073	1.10	0.54094	-1.03	0.92197	Susd4

-3.40	0.00034	-3.04	0.00703	-1.28	0.27476	-1.15	0.55796	Cyp4a12b
-3.43	0.00133	-4.17	0.00443	1.01	0.97097	-1.21	0.58428	Cyp7b1
-3.43	0.00285	-3.81	0.02495	-1.64	0.42339	-1.82	0.33654	Serpina4-ps1
-3.45	0.00083	-3.43	0.05993	1.33	0.33574	1.34	0.53315	C4a
-3.57	0.00160	-4.34	0.00048	1.13	0.55372	-1.07	0.74539	1700080G11Rik
-3.69	0.00055	-4.00	0.00005	-1.03	0.74885	-1.12	0.55980	Tlr5
-3.73	0.00222	-2.11	0.02672	1.06	0.76289	1.88	0.07889	Trhde
-3.78	0.00285	-4.22	0.02452	1.81	0.04302	1.62	0.35234	Lrtm1
-3.79	0.00291	-3.99	0.00265	-1.06	0.64762	-1.12	0.66960	Cyp2c37
-4.38	0.00331	-5.30	0.00076	1.33	0.40945	1.10	0.82779	Hsd3b5
-4.76	0.00027	-6.96	0.00502	1.29	0.64219	-1.14	0.87341	Elovl3
-5.28	0.00627	-5.82	0.00634	1.28	0.15152	1.16	0.76192	Etnppl
-5.52	0.00024	-5.99	0.00122	1.18	0.59656	1.08	0.71746	Orm3
-5.62	0.00273	-5.58	0.00894	1.05	0.89929	1.06	0.89166	Gm23340
-6.99	0.00100	-11.72	0.01137	1.08	0.90679	-1.56	0.48703	Obp2a

Supplementary Table 3: Proteins with a role in lipid metabolism were analyzed by immunoblot in the normal tissues (NT) and the tumor tissues (TT) of Control-AAV and Chemerin-156-AAV infected mice. ApoA1 and Fabp5 were similarly changed in the tumors of both groups. ** $p < 0.01$, *** $p < 0.001$.

Protein	Animal Group	Tissue	Animal number	Median and range	p-value
ApoA1	Control-AAV	NT	9	0.9 (0.0 - 1.4)	
	Control-AAV	TT	9	0.1 (0.0 - 0.5)	**
	Chemerin-156-AAV	NT	12	0.9 (0.3 - 1.8)	
	Chemerin-156-AAV	TT	12	0.0 (0.0 - 0.4)	***
Fabp5	Control-AAV	NT	9	0.2 (0.0 - 0.6)	
	Control-AAV	TT	9	0.4 (0.3 - 2.0)	
	Chemerin-156-AAV	NT	12	0.2 (0.0 - 0.5)	
	Chemerin-156-AAV	TT	12	0.9 (0.3 - 1.7)	***
FAS	Control-AAV	NT	9	0.3 (0.2 - 1.3)	
	Control-AAV	TT	9	0.8 (0.4 - 1.3)	
	Chemerin-156-AAV	NT	12	0.7 (0.2 - 2.5)	
	Chemerin-156-AAV	TT	12	1.0 (0.3 - 2.4)	
SREBP2 Active	Control-AAV	NT	9	0.8 (0.2 - 2.5)	
	Control-AAV	TT	9	0.5 (0.2 - 1.0)	
	Chemerin-156-AAV	NT	12	0.8 (0.3 - 1.8)	
	Chemerin-156-AAV	TT	12	0.3 (0.0 - 1.7)	
SREBP2 Full-length	Control-AAV	NT	9	0.4 (0.1 - 1.0)	
	Control-AAV	TT	9	0.7 (0.2 - 1.2)	
	Chemerin-156-AAV	NT	12	0.4 (0.1 - 1.4)	
	Chemerin-156-AAV	TT	12	0.5 (0.0 - 1.5)	
SND1	Control-AAV	NT	9	0.9 (0.5 - 1.1)	
	Control-AAV	TT	8	0.9 (0.6 - 1.7)	
	Chemerin-156-AAV	NT	12	0.8 (0.5 - 1.4)	
	Chemerin-156-AAV	TT	11	0.9 (0.6 - 2.4)	
SREBP1c Active	Control-AAV	NT	9	0.4 (0.0 - 1.1)	
	Control-AAV	TT	8	0.6 (0.1 -2.0)	
	Chemerin-156-AAV	NT	12	0.3 (0.0 - 1.8)	
	Chemerin-156-AAV	TT	12	0.4 (0.0 - 1.8)	
SREBP1c Full-length	Control-AAV	NT	9	0.5 (0.1 - 1.2)	
	Control-AAV	TT	9	0.7 (0.2 - 2.1)	
	Chemerin-156-AAV	NT	12	0.7 (0.2 - 2.0)	
	Chemerin-156-AAV	TT	12	0.9 (0.3 - 1.9)	
SCD1	Control-AAV	NT	9	0.8 (0.0 - 1.8)	
	Control-AAV	TT	9	1.0 (0.0 -1.8)	
	Chemerin-156-AAV	NT	12	0.6 (0.0 - 1.7)	
	Chemerin-156-AAV	TT	12	0.6 (0.0 - 1.5)	

Supplementary Table 4: Triglyceride (TG) species in normal tissue (NT) and tumor tissue (TT) of Control-AAV and Chemerin-156-AAV injected mice. Lipids are given in nmol/mg wet weight. Hepatic triglyceride species were increased in the tumors but did neither differ in NT nor TT of Control-AAV and Chemerin-156-AAV injected mice. * $p < 0.05$, ** $p < 0.01$, *** $p < 0.001$.

TG Species	Animal Group	Tissue	Median and range	p-value (relative to the respective NT)
48:0	Control-AAV	NT	0.01 (0.01 - 0.02)	
	Control-AAV	TT	0.04 (0.02 - 0.14)	**
	Chemerin-156-AAV	NT	0.01 (0.01 - 0.01)	
	Chemerin-156-AAV	TT	0.06 (0.01 -0.18)	**
48:1	Control-AAV	NT	0.01 (0.01 - 0.03)	
	Control-AAV	TT	0.15 (0.04 - 0.38)	**
	Chemerin-156-AAV	NT	0.01 (0.01 - 0.02)	
	Chemerin-156-AAV	TT	0.13 (0.02 - 0.41)	***
48:2	Control-AAV	NT	0.01 (0.00 - 0.04)	
	Control-AAV	TT	0.13 (0.03 - 0.31)	**
	Chemerin-156-AAV	NT	0.01 (0.00 - 0.04)	
	Chemerin-156-AAV	TT	0.1 (0.02 - 0.36)	***
48:3	Control-AAV	NT	0.01 (0.00 - 0.30)	
	Control-AAV	TT	0.05 (0.02 - 0.18)	**
	Chemerin-156-AAV	NT	0.00 (0.00 - 0.03)	
	Chemerin-156-AAV	TT	0.05 (0.00 - 0.16)	**
49:1	Control-AAV	NT	0.00 (0.00 - 0.00)	
	Control AAV	TT	0.02 (0.00 - 0.04)	**
	Chemerin-156-AAV	NT	0.00 (0.00 - 0.00)	
	Chemerin-156-AAV	TT	0.01 (0.00 - 0.05)	***
49:2	Control-AAV	NT	0.00 (0.00 - 0.01)	
	Control-AAV	TT	0.00 (0.00 - 0.01)	***
	Chemerin-156-AAV	NT	0.02 (0.00 - 0.06)	
	Chemerin-156-AAV	TT	0.02 (0.00 - 0.06)	***
50:1	Control-AAV	NT	0.08 (0.04 - 0.23)	
	Control-AAV	TT	1.38 (0.39 - 4.09)	**
	Chemerin-156-AAV	NT	0.09 (0.06 - 0.17)	
	Chemerin-156-AAV	TT	1.48 (0.19 - 3.61)	***
50:2	Control-AAV	NT	0.12 (0.05 - 0.41)	
	Control-AAV	TT	2.29 (0.65 - 6.11)	***
	Chemerin-156-AAV	NT	0.14 (0.06 – 0.48)	
	Chemerin-156-AAV	TT	1.95 (0.21 - 5.59)	***
50:3	Control-AAV	NT	0.08 (0.03 - 0.36)	
	Control-AAV	TT	1.00 (0.30 - 2.87)	***
	Chemerin-156-AAV	NT	0.07 (0.03 – 0.45)	
	Chemerin-156-AAV	TT	0.90 (0.08 - 2.90)	**
50:4	Control-AAV	NT	0.03 (0.01 - 0.15)	
	Control-AAV	TT	0.22 (0.08 - 0.71)	**
	Chemerin-156-AAV	NT	0.02 (0.01 - 0.19)	
	Chemerin-156-AAV	TT	0.24 (0.02 - 0.72)	**
50:5	Control-AAV	NT	0.01 (0.00 – 0.04)	
	Control-AAV	TT	0.05 (0.02 - 0.23)	**
	Chemerin-156-AAV	NT	0.00 (0.00 -0.05)	
	Chemerin-156-AAV	TT	0.05 (0.00 - 0.19)	*
50:6	Control-AAV	NT	0.00 (0.00 - 0.01)	
	Control-AAV	TT	0.01 (0.00 - 0.07)	*
	Chemerin-156-AAV	NT	0.00 (0.00 - 0.01)	

	Chemerin-156-AAV	TT	0.01 (0.00 - 0.06)	
51:1	Control-AAV	NT	0.00 (0.00 - 0.00)	
	Control-AAV	TT	0.02 (0.01 - 0.05)	***
	Chemerin-156-AAV	NT	0.00 (0.00 - 0.00)	
	Chemerin-156-AAV	TT	0.02 (0.00 - 0.05)	***
51:2	Control-AAV	NT	0.01 (0.00 - 0.02)	
	Control-AAV	TT	0.11 (0.03 - 0.28)	***
	Chemerin-156-AAV	NT	0.01 (0.00 - 0.02)	
	Chemerin-156-AAV	TT	0.10 (0.01 - 0.25)	***
51:3	Control-AAV	NT	0.01 (0.00 -0.04)	
	Control-AAV	TT	0.09 (0.03 - 0.25)	***
	Chemerin-156-AAV	NT	0.01 (0.00 - 0.05)	
	Chemerin-156-AAV	TT	0.09 (0.01 - 0.23)	***
51:4	Control-AAV	NT	0.01 (0.00 – 0.03)	
	Control-AAV	TT	0.03 (0.01 - 0.09)	**
	Chemerin-156-AAV	NT	0.01 (0.00 -0.04)	
	Chemerin-156-AAV	TT	0.04 (0.00 - 0.10)	*
52:2	Control-AAV	NT	0.35 (0.18 - 1.17)	
	Control-AAV	TT	7.44 (1.98 - 22.39)	***
	Chemerin-156-AAV	NT	0.45 (0.19 - 1.19)	
	Chemerin-156-AAV	TT	6.67 (0.70 - 15.78)	***
52:3	Control-AAV	NT	0.78 (0.34 - 3.30)	
	Control-AAV	TT	9.02 (3.64 - 28.14)	***
	Chemerin-156-AAV	NT	0.87 (0.36 - 4.15)	
	Chemerin-156-AAV	TT	9.50 (0.90 - 21.60)	***
52:4	Control-AAV	NT	0.69 (0.29 - 3.12)	
	Control-AAV	TT	3.70 (1.65 - 11.95)	**
	Chemerin-156-AAV	NT	0.65 (0.26 - 4.21)	
	Chemerin-156-AAV	TT	4.36 (0.46 - 10.98)	**
52:5	Control-AAV	NT	0.14 (0.04 - 0.70)	
	Control-AAV	TT	0.78 (0.33 - 2.36)	**
	Chemerin-156-AAV	NT	0.11 (0.04 -0.91)	
	Chemerin-156-AAV	TT	0.86 (0.08 - 2.46)	**
52:6	Control-AAV	NT	0.02 (0.01 - 0.13)	
	Control-AAV	TT	0.15 (0.06 - 0.55)	**
	Chemerin-156-AAV	NT	0.02 (0.01 - 0.17)	
	Chemerin-156-AAV	TT	0.14 (0.02 - 0.51)	*
52:7	Control-AAV	NT	0.00 (0.00 - 0.03)	
	Control-AAV	TT	0.03 (0.01 - 0.14)	**
	Chemerin-156-AAV	NT	0.01 (0.00 -0.03)	
	Chemerin-156-AAV	TT	0.03 (0.00 - 0.12)	*
53:2	Control-AAV	NT	0.00 (0.00 - 0.01)	
	Control-AAV	TT	0.08 (0.03 - 0.24)	***
	Chemerin-156-AAV	NT	0.00 (0.00 - 0.01)	
	Chemerin-156-AAV	TT	0.08 (0.01 - 0.17)	***
53:3	Control-AAV	NT	0.01 (0.01 - 0.04)	
	Control-AAV	TT	0.15 (0.05 - 0.42)	***
	Chemerin-156-AAV	NT	0.01 (0.01 - 0.05)	
	Chemerin-156-AAV	TT	0.14 (0.01 - 0.32)	***
53:4	Control-AAV	NT	0.01 (0.00 - 0.05)	
	Control-AAV	TT	0.10 (0.04 - 0.29)	***
	Chemerin-156-AAV	NT	0.01 (0.00 -0.07)	
	Chemerin-156-AAV	TT	0.12 (0.01 - 0.26)	***
53:5	Control-AAV	NT	0.01 (0.00 - 0.03)	
	Control-AAV	TT	0.07 (0.03 - 0.15)	***
	Chemerin-156-AAV	NT	0.01 (0.00 - 0.05)	
	Chemerin-156-AAV	TT	0.07 (0.01 - 0.16)	***

54:3	Control-AAV	NT	0.25 (0.13 - 0.74)	
	Control-AAV	TT	5.14 (1.38 - 18.52)	***
	Chemerin-156-AAV	NT	0.29 (0.14 - 0.75)	
	Chemerin-156-AAV	TT	5.67 (0.53 - 10.50)	***
54:4	Control-AAV	NT	0.53 (0.28 - 1.78)	
	Control-AAV	TT	6.78 (2.42 - 22.65)	***
	Chemerin-156-AAV	NT	0.56 (0.28 - 2.19)	
	Chemerin-156-AAV	TT	7.48 (0.75 - 14.23)	***
54:5	Control-AAV	NT	0.62 (0.31 - 2.20)	
	Control-AAV	TT	4.36 (2.35 - 12.76)	***
	Chemerin-156-AAV	NT	0.54 (0.27 - 2.93)	
	Chemerin-156-AAV	TT	4.39 (0.63 - 10.26)	***
54:6	Control-AAV	NT	0.38 (0.16 - 1.42)	
	Control-AAV	TT	2.04 (1.07 - 5.17)	***
	Chemerin-156-AAV	NT	0.32 (0.14 - 1.92)	
	Chemerin-156-AAV	TT	1.85 (0.35 - 5.34)	**
54:7	Control-AAV	NT	0.07 (0.02 - 0.36)	
	Control-AAV	TT	0.45 (0.22 - 1.20)	**
	Chemerin-156-AAV	NT	0.06 (0.02 - 0.50)	
	Chemerin-156-AAV	TT	0.41 (0.06 - 1.39)	**
55:2	Control-AAV	NT	0.00 (0.00 - 0.00)	
	Control-AAV	TT	0.01 (0.00 - 0.04)	**
	Chemerin-156-AAV	NT	0.00 (0.00 - 0.00)	
	Chemerin-156-AAV	TT	0.01 (0.00 - 0.02)	**
55:3	Control-AAV	NT	0.00 (0.00 - 0.01)	
	Control-AAV	TT	0.06 (0.02 - 0.20)	***
	Chemerin-156-AAV	NT	0.00 (0.00 - 0.01)	
	Chemerin-156-AAV	TT	0.06 (0.00 - 0.12)	***
55:4	Control-AAV	NT	0.00 (0.00 - 0.02)	
	Control-AAV	TT	0.07 (0.02 - 0.20)	***
	Chemerin-156-AAV	NT	0.00 (0.00 - 0.02)	
	Chemerin-156-AAV	TT	0.07 (0.01 - 0.13)	***
55:5	Control-AAV	NT	0.00 (0.00 - 0.02)	
	Control-AAV	TT	0.05 (0.02 - 0.15)	***
	Chemerin-156-AAV	NT	0.00 (0.00 - 0.02)	
	Chemerin-156-AAV	TT	0.05 (0.01 - 0.12)	***
56:3	Control-AAV	NT	0.02 (0.02 - 0.08)	
	Control-AAV	TT	0.52 (0.13 - 2.43)	***
	Chemerin-156-AAV	NT	0.03 (0.01 - 0.06)	
	Chemerin-156-AAV	TT	0.67 (0.06 - 0.95)	**
56:4	Control-AAV	NT	0.07 (0.05 - 0.16)	
	Control-AAV	TT	0.98 (0.24 - 3.64)	***
	Chemerin-156-AAV	NT	0.08 (0.04 - 0.16)	
	Chemerin-156-AAV	TT	0.97 (0.15 - 1.72)	***
56:5	Control-AAV	NT	0.15 (0.09 - 0.32)	
	Control-AAV	TT	1.50 (0.47 - 4.80)	***
	Chemerin-156-AAV	NT	0.17 (0.09 - 0.40)	
	Chemerin-156-AAV	TT	1.70 (0.40 - 3.22)	***
56:6	Control-AAV	NT	0.24 (0.12 - 0.61)	
	Control-AAV	TT	2.20 (0.84 - 5.94)	***
	Chemerin-156-AAV	NT	0.22 (0.11 - 0.71)	
	Chemerin-156-AAV	TT	2.18 (0.54 - 4.75)	***
56:7	Control-AAV	NT	0.33 (0.14 - 0.95)	
	Control-AAV	TT	2.75 (1.20 - 6.82)	***
	Chemerin-156-AAV	NT	0.26 (0.13 - 1.12)	
	Chemerin-156-AAV	TT	2.53 (0.56 - 6.07)	***
56:8	Control-AAV	NT	0.26 (0.11 - 0.85)	

	Control-AAV	TT	1.60 (0.86 - 3.52)	***
	Chemerin-156-AAV	NT	0.21 (0.10 - 1.11)	
	Chemerin-156-AAV	TT	1.25 (0.26 - 3.29)	**
57:4	Control-AAV	NT	0.00 (0.00 - 0.00)	
	Control-AAV	TT	0.01 (0.00 - 0.04)	**
	Chemerin-156-AAV	NT	0.00 (0.00 - 0.00)	
	Chemerin-156-AAV	TT	0.01 (0.00 - 0.01)	*
57:5	Control-AAV	NT	0.00 (0.00 - 0.00)	
	Control-AAV	TT	0.02 (0.00 - 0.05)	***
	Chemerin-156-AAV	NT	0.00 (0.00 - 0.00)	
	Chemerin-156-AAV	TT	0.02 (0.00 - 0.03)	***
58:3	Control-AAV	NT	0.01 (0.01 - 0.01)	
	Control-AAV	TT	0.05 (0.01 - 0.19)	***
	Chemerin-156-AAV	NT	0.01 (0.00 - 0.01)	
	Chemerin-156-AAV	TT	0.05 (0.01 - 0.07)	**
58:5	Control-AAV	NT	0.02 (0.01 - 0.03)	
	Control-AAV	TT	0.16 (0.03 - 0.64)	***
	Chemerin-156-AAV	NT	0.02 (0.01 - 0.04)	
	Chemerin-156-AAV	TT	0.16 (0.06 - 0.33)	**
58:6	Control-AAV	NT	0.05 (0.03 - 0.08)	
	Control-AAV	TT	0.05 (0.13 - 1.79)	***
	Chemerin-156-AAV	NT	0.05 (0.03 - 0.10)	
	Chemerin-156-AAV	TT	0.06 (0.17 - 1.11)	***
58:7	Control-AAV	NT	0.10 (0.05 - 0.20)	
	Control-AAV	TT	1.01 (0.30 - 2.74)	***
	Chemerin-156-AAV	NT	0.08 (0.04 - 0.22)	
	Chemerin-156-AAV	TT	0.90 (0.28 - 2.00)	***
58:8	Control-AAV	NT	0.16 (0.08 - 0.35)	
	Control-AAV	TT	1.26 (0.45 - 3.36)	***
	Chemerin-156-AAV	NT	0.13 (0.07 - 0.42)	
	Chemerin-156-AAV	TT	1.27 (0.34 - 2.61)	***
59:8	Control-AAV	NT	0.00 (0.00 - 0.00)	
	Control-AAV	TT	0.01 (0.00 - 0.03)	***
	Chemerin-156-AAV	NT	0.00 (0.00 - 0.00)	
	Chemerin-156-AAV	TT	0.01 (0.00 - 0.02)	***
60:7	Control-AAV	NT	0.01 (0.01 - 0.02)	
	Control-AAV	TT	0.17 (0.02 - 0.73)	***
	Chemerin-156-AAV	NT	0.01 (0.00 - 0.02)	
	Chemerin-156-AAV	TT	0.15 (0.04 - 0.47)	**
60:8	Control-AAV	NT	0.03 (0.02 - 0.04)	
	Control-AAV	TT	0.31 (0.07 - 0.10)	***
	Chemerin-156-AAV	NT	0.02 (0.01 - 0.07)	
	Chemerin-156-AAV	TT	0.30 (0.10 - 0.78)	***
60:9	Control-AAV	NT	0.03 (0.02 – 0.05)	
	Control-AAV	TT	0.32 (0.10 - 0.85)	***
	Chemerin-156-AAV	NT	0.03 (0.01 - 0.07)	
	Chemerin-156-AAV	TT	0.25 (0.10 - 0.74)	***

Supplementary Table 5: Diacylglycerol (DG) species in the normal tissues (NT) and the tumor tissues (TT) of Control-AAV and Chemerin-156-AAV infected mice. Lipids are given in nmol/mg wet weight. Hepatic DG species mostly increased in the tumors. DG species were similarly concentrated in tumorous and non-tumorous tissues of Control-AAV and Chemerin-156-AAV injected mice. * $p < 0.05$, ** $p < 0.01$, *** $p < 0.001$.

DG Species	Animal Group	Tissue	Median and range	p-value (relative to the respective NT)
32:0	Control-AAV	NT	0.02 (0.01 - 0.02)	
	Control-AAV	TT	0.04 (0.01 - 0.07)	***
	Chemerin-156-AAV	NT	0.02 (0.01 - 0.02)	
	Chemerin-156-AAV	TT	0.05 (0.02 -0.06)	***
32:1	Control-AAV	NT	0.01 (0.01 - 0.02)	
	Control-AAV	TT	0.04 (0.01 - 0.07)	***
	Chemerin-156-AAV	NT	0.01 (0.01 - 0.02)	
	Chemerin-156-AAV	TT	0.05 (0.02 - 0.07)	***
34:1	Control-AAV	NT	0.11 (0.09 - 0.18)	
	Control-AAV	TT	0.50 (0.16 - 1.07)	***
	Chemerin-156-AAV	NT	0.13 (0.10 - 0.19)	
	Chemerin-156-AAV	TT	0.58 (0.20 - 0.75)	***
34:2	Control-AAV	NT	0.17 (0.11 - 0.41)	
	Control-AAV	TT	0.61 (0.26 - 1.15)	***
	Chemerin-156-AAV	NT	0.22 (0.16 - 0.41)	
	Chemerin-156-AAV	TT	0.68 (0.21 - 0.95)	***
34:3	Control-AAV	NT	0.03 (0.01 - 0.08)	
	Control-AAV	TT	0.12 (0.04 - 0.25)	**
	Chemerin-156-AAV	NT	0.04 (0.03 - 0.09)	
	Chemerin-156-AAV	TT	0.14 (0.03 - 0.24)	**
36:2	Control-AAV	NT	0.11 (0.08 - 0.23)	
	Control-AAV	TT	0.50 (0.13 - 0.91)	***
	Chemerin-156-AAV	NT	0.16 (0.10 – 0.23)	
	Chemerin-156-AAV	TT	0.51 (0.14 – 1.07)	***
36:3	Control-AAV	NT	0.28 (0.12 - 0.58)	
	Control-AAV	TT	0.67 (0.23 - 1.11)	*
	Chemerin-156-AAV	NT	0.31 (0.15 – 0.64)	
	Chemerin-156-AAV	TT	0.80 (0.17 - 1.45)	*
36:4	Control-AAV	NT	0.25 (0.12 - 0.64)	
	Control-AAV	TT	0.43 (0.21 - 0.78)	
	Chemerin-156-AAV	NT	0.31 (0.15 - 0.64)	
	Chemerin-156-AAV	TT	0.45 (0.14 - 0.80)	
36:5	Control-AAV	NT	0.02 (0.01 - 0.08)	
	Control-AAV	TT	0.03 (0.01 - 0.12)	
	Chemerin-156-AAV	NT	0.03 (0.02 - 0.08)	
	Chemerin-156-AAV	TT	0.04 (0.01 - 0.14)	
38:3	Control-AAV	NT	0.01 (0.00 - 0.03)	
	Control-AAV	TT	0.04 (0.01 - 0.11)	***
	Chemerin-156-AAV	NT	0.02 (0.01 - 0.03)	
	Chemerin-156-AAV	TT	0.05 (0.02 - 0.12)	***
38:4	Control-AAV	NT	0.16 (0.09 - 0.24)	
	Control-AAV	TT	0.21 (0.10 - 0.39)	
	Chemerin-156-AAV	NT	0.12 (0.07 - 0.17)	
	Chemerin-156-AAV	TT	0.23 (0.20 - 0.42)	***
38:5	Control-AAV	NT	0.07 (0.04 - 0.14)	
	Control-AAV	TT	0.54 (0.21 - 1.48)	***
	Chemerin-156-AAV	NT	0.08 (0.04 - 0.12)	

	Chemerin-156-AAV	TT	0.51 (0.15 - 0.98)	***
38:6	Control-AAV	NT	0.10 (0.06 - 0.17)	
	Control-AAV	TT	0.54 (0.21 - 1.48)	***
	Chemerin-156-AAV	NT	0.11 (0.07 - 0.17)	
	Chemerin-156-AAV	TT	0.51 (0.15 - 0.98)	***
38:7	Control-AAV	NT	0.00 (0.00 – 0.02)	
	Control-AAV	TT	0.03 (0.01 - 0.06)	***
	Chemerin-156-AAV	NT	0.00 (0.00 -0.02)	
	Chemerin-156-AAV	TT	0.03 (0.00 - 0.06)	***
40:5	Control-AAV	NT	0.00 (0.00 - 0.03)	
	Control-AAV	TT	0.03 (0.00 - 0.09)	**
	Chemerin-156-AAV	NT	0.01 (0.00 - 0.02)	
	Chemerin-156-AAV	TT	0.04 (0.01 - 0.11)	*
40:6	Control-AAV	NT	0.03 (0.02 – 0.07)	
	Control-AAV	TT	0.11 (0.03 - 0.23)	***
	Chemerin-156-AAV	NT	0.04 (0.02 - 0.05)	
	Chemerin-156-AAV	TT	0.11 (0.04 - 0.29)	***
40:7	Control-AAV	NT	0.05 (0.03 - 0.12)	
	Control-AAV	TT	0.18 (0.05 – 0.37)	**
	Chemerin-156-AAV	NT	0.08 (0.03 - 0.13)	
	Chemerin-156-AAV	TT	0.20 (0.05 - 0.56)	**
40:8	Control-AAV	NT	0.07 (0.03 - 0.16)	
	Control-AAV	TT	0.11 (0.04 - 0.24)	
	Chemerin-156-AAV	NT	0.09 (0.02 - 0.20)	
	Chemerin-156-AAV	TT	0.11 (0.02 - 0.34)	

Supplementary Table 6: Antibodies used for immunblot experiments. The table provides information about the respective companies where the reagent were ordered, the order number and the dilution of the antibodies for immunoblot experiments.

Antigen	Company	Order number	Dilution
ApoA1	Biodesign	K45252G	1 : 20.000
β-catenin	Cell Signaling	2698	1 : 1.000
Chemerin	R&D Systems	AF2325	1 : 500
CMKLR1	Novus Biologicals	NBP2-13847	1 : 500
Fabp5	R&D Systems	AF1476	1 : 5.000
FAS	Cell Signaling	3180	1 : 1.000
GAPDH	Cell Signaling	2118	1 : 1.000
MnSOD	Lab Frontier	LF-PA0021	1 : 2.000
phospho-ß-Catenin (Ser33/37/Thr41)	Cell Signaling	9561	1 : 1.000
phospho-ß-Catenin (Ser552)	Cell Signaling	9566	1 : 1.000
SCD1	Cell Signaling	2794	1 : 1.000
SND1	Signalway Antibody	38700	1 : 400
SREBP1c	Thermo Scientific	MS-1207-P1	1 : 1.000
SREBP2	Cayman	10007663	1 : 1.000

Supplementary Table 7: Primers used for real-time RT-PCR analysis. Cyclophilin A (Cyclo) was used for normalization.

Gene	Sequence uni-Primer 5′ → 3′	Sequence rev-Primer 5′ → 3′
**Alox5**	GCATGCAGCTGTAAACTTCG	CCAGTGAAGGAAGCCATGAT
**CD38**	ACGCTGCCTCATCTACACTC	GGGGCGTAGTCTTCTCTTGT
**Chemerin**	AAACACCCACCTGTGCAGT	TTTTACCCTTGGGGTCCATT
**CMKLR1**	CTTCTCCCCTAATCCCCTCA	GGGGTGAGTGAGCCATTTT
**Col4a3**	ACGTGATGGAATTCCTGGTC	GTTCCAGGCTTGTCTGGC
**Egr1**	CCTATGAGCACCTGACCACA	GACCAATCCTCCGACCTCTT
**F4/80**	TGCTCTTCCTGATGGTGAGA	CCCCGTCTCTGTATTCAACC
**Fabp5**	ACGGCTTTGAGGAGTACATGA	GTCTGCACCTTCCAAGACG
**G6PC**	CCTGTGAGACCGGACCAG	TTGCTGTGGCTGAAACTTTCAG
**HMG-CoA-R**	TGTGCTTGGGGCTTCTGTA	CCACGTTCATGAGTTTCCATT
**IL-6**	CTCTGGGAAATCGTGGAAAT	CCAGTTTGGTAGCATCCATC
**Ly49C**	CCCTATTCCAGGGAGCTGT	TCTGTTTACCAGGAAGGAAGATG
**Ncr1**	TTGGCTCTTACAACGACTATGC	GTTGAAAGGTCAAACTCCCAAT
**Pnpla5**	ACCATTGCCGACAAGGTTAC	CACTGAGAAAGGCATGCAAG
**Slc12a1**	TATTTGCACAAACGGAGTGGTG	CTCAAGTCATTCTCCTGGTCATT
**Spink1**	CACCCAGATCTTCGACAATG	GTCCTCATTCGAAAAGGTGG
**αSMA**	CCAGCACCATGAAGATCAAG	CTTCGTCGTATTCCTGTTTGC

Supplementary figure 1

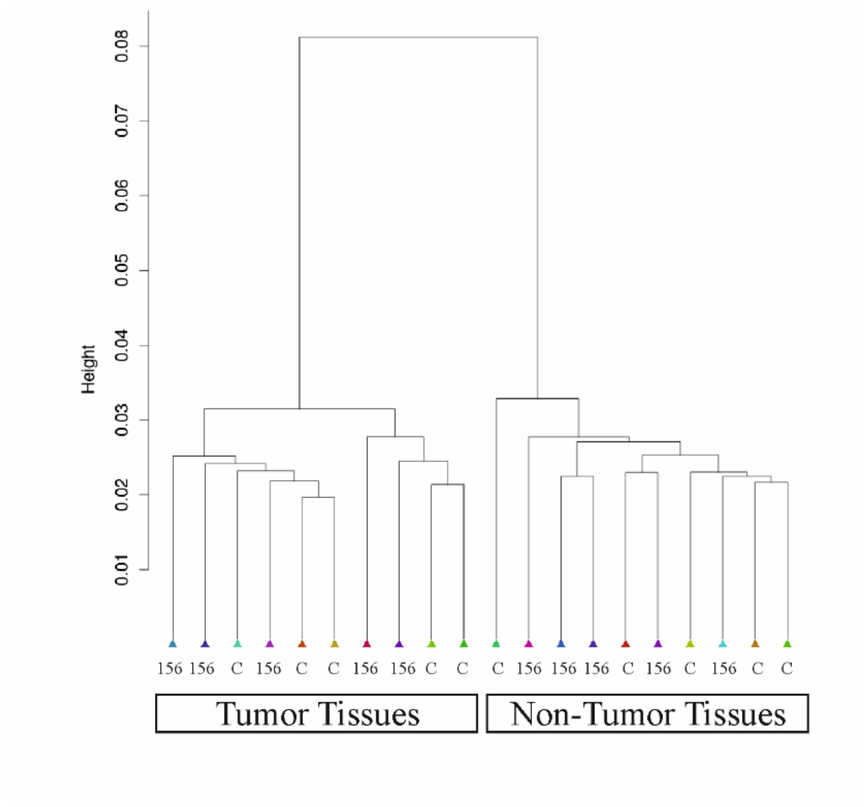

Cluster dendrogram of the microarray data. Normal tissues are more similar to each other than they are to the tumor tissues. Tissues of Control-AAV (C) and Chemerin-156-AAV (156) infected mice are similar to each other.
